# High level of circulating vitamin D during neoadjuvant therapy may lower risk of metastatic progression in high-risk rectal cancer

**DOI:** 10.1186/s12885-019-5724-z

**Published:** 2019-05-23

**Authors:** Hanna Abrahamsson, Alina C. Porojnicu, Jonas C. Lindstrøm, Svein Dueland, Kjersti Flatmark, Knut H. Hole, Therese Seierstad, Johan Moan, Kathrine Røe Redalen, Sebastian Meltzer, Anne Hansen Ree

**Affiliations:** 1 Department of Oncology, Akershus University Hospital, P.O. Box 1000, 1478 Lørenskog, Norway; 2 Institute of Clinical Medicine, University of Oslo, Oslo, Norway; 3 Department of Oncology, Vestre Viken Hospital Trust, Drammen, Norway; 4 Health Services Research Center, Akershus University Hospital, Lørenskog, Norway; 5 Department of Oncology, Oslo University Hospital, Oslo, Norway; 6 Department of Gastroenterological Surgery, Oslo University Hospital, Oslo, Norway; 7 Department of Tumor Biology, Oslo University Hospital, Oslo, Norway; 8 Department of Radiology, Oslo University Hospital, Oslo, Norway; 9 Department of Radiation Oncology, Oslo University Hospital, Oslo, Norway; 10 Institute of Physics, University of Oslo, Oslo, Norway; 11 Department of Physics, Norwegian University of Science and Technology, Trondheim, Norway

**Keywords:** Vitamin D, Rectal cancer, Neoadjuvant, Chemotherapy, Radiotherapy, Metastasis

## Abstract

**Background:**

Following curative-intent neoadjuvant therapy in locally advanced rectal cancer, metastatic progression is still dominant. We investigated if patients’ circulating 25-hydroxyvitamin D [25(OH)D] levels were associated with outcome.

**Methods:**

Serum 25(OH)D concentration was assessed by liquid chromatography-mass spectrometry in samples collected from 84 patients at baseline, completion of the neoadjuvant therapy, and treatment evaluation before surgery, and analyzed with respect to season, disease presentation, and treatment effects.

**Results:**

In the cohort of patients residing at latitude 58–62°N, baseline 25(OH)D differed significantly over the seasons, with highest measures (mean of 71.2 ± 5.6 nmol/L) in summer and lowest (48.7 ± 4.5 nmol/L) in spring, and changed over the three-month neoadjuvant period till response evaluation solely owing to season. The patient subgroup with slightly reduced performance status, anemia, and T4 disease that did not respond to the neoadjuvant therapy (ypT4 cases), had significantly lower baseline 25(OH)D (below 50 nmol/L) than T4 cases with response (ypT0–3) and T2–3 cases (above 60 nmol/L). Compared to the T4 patients with levels above 50 nmol/L, regarded as sufficient for a healthy bone status, those presenting levels below had significantly heightened risk of disease progression (mainly metastasis) and death, with hazard ratio of 3 and 17, respectively, on adjustment for age, sex, body mass index, and season.

**Conclusion:**

Rectal cancer T4 cases had high risk of metastatic progression and death if circulating 25(OH)D levels were insufficient but obtained short-term and long-term outcome to neoadjuvant treatment no worse than patients with T2–3 disease when 25(OH)D was sufficient.

**Trial registration:**

ClinicalTrials.gov NCT00278694; registration date: 16 January 2006, retrospective to enrollment of the first 10 patients of the current report.

## Background

With reference to the demography of colorectal cancer (CRC), principally owing to an aging population, the incidence in the Nordic countries is among the highest in the world [1]. It has been hypothesized that high incidence and poor prognosis may partly be attributed to an insufficient vitamin D status [2–4]. Circulating vitamin D is strongly associated with exposure to solar ultraviolet radiation [5], which in the Nordic countries (mainland latitude 55-72^o^N) results in significant variation over the year [6, 7], especially if the diet is scarce in supplementary vitamin D in calendar months when the basic requirement cannot be met by sun exposure alone [8]. In both northern and southern regions of Norway the prognosis of CRC across all disease stages seems to be better when diagnosed in summer and fall compared to winter and spring [9, 10].

Exposure to sunlight causes conversion of 7-dehydrocholesterol in the skin to cholecalciferol (vitamin D_3_), which is further metabolized by two-step hydroxylation via 25-hydroxycholecalciferol [25-hydroxyvitamin D_3_ (25(OH)D_3_)] to obtain the hormonally active form of vitamin D_3_, calcitriol (1,25-dihydroxyvitamin D_3_). This metabolite binds to the vitamin D receptor and turns it into an active transcription factor. In addition to its classic functions in the calcium homeostasis, a discrete number of target genes are implicated in biological processes that counteract malignant progression, such as cell cycle control, DNA damage repair, and apoptosis [11]. According to the Nordic Nutrition Recommendations, a serum level of 25(OH)D (endogenous and supplementary by diet) of 50 nmol/L is adequate to maintain healthy bone status [12], though it has been suggested that the optimum serum concentration for multiple health outcomes may be higher [13, 14]. Of note, vitamin D sufficiency may be protective against development of inflammatory bowel disease [15] and accordingly, deficient circulating levels are associated with heightened risk of CRC development in these patients [16], supporting an association between vitamin D, inflammation, and CRC.

**Fig. 1:**
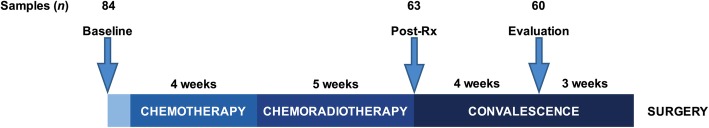
The timing of serum sampling (arrows) within the neoadjuvant treatment protocol. *Abbreviations: n* the numbers of samples at each sampling point, *Post-Rx* completion of neoadjuvant therapy

Patients with locally advanced rectal cancer (LARC), typically presenting a primary tumor that grows either beyond the rectal wall (T3 disease), into neighboring pelvic organs (T4 disease), or with local lymph node involvement, need to undergo neoadjuvant therapy before radical surgery is feasible. Histologic ypTN status of the surgical specimen is a surrogate marker for response to the neoadjuvant therapy in lieu of progression-free and overall survival (PFS, OS) as clinical endpoints. Patients with T4 disease are at particularly high risk of poor treatment outcome [17]. In a prospective LARC study [18], patients with mainly T3–4 and N-positive disease were given 4 weeks of induction chemotherapy and 5 weeks of sequential pelvic chemoradiotherapy before they proceeded to surgery approximately 7 weeks after completion of the neoadjuvant therapy (Fig. 1). ypTN status was determined and patients were followed to record recurrent disease and long-term survival.

To our knowledge, there are no previous reports on vitamin D status and outcome of combined-modality therapy in rectal cancer. In this post hoc analysis, we assessed circulating levels of 25(OH)D, which in clinical practice are used as measure of the vitamin D status [19], at the time of diagnosis (termed baseline), at completion of the neoadjuvant therapy (termed post-Rx), and approximately 3 weeks before surgery (termed evaluation) in our study cohort (Fig. 1) and investigated if there were any associations with the season of diagnosis, disease presentation, and treatment effects.

## Methods

### Patients and treatment

Patient eligibility criteria of the LARC study (ClinicalTrials.gov NCT00278694) have been described previously [18]. Patients had to be Eastern Cooperative Oncology Group (ECOG) performance status 0 or 1. Ninety-seven patients were prospectively enrolled between 5 October 2005 and 3 March 2010 [18]. Within the current spin-off study, the 84 cases population excluded 10 patients with metastatic disease at presentation and three for whom serum 25(OH)D measurement failed. The neoadjuvant protocol has been detailed previously [18]. A preoperative evaluation of the neoadjuvant treatment was undertaken at a median of 31 (range, 23–43) days after its completion, and surgery was done at a median of 21 (range, 7–41) days thereafter. In accordance with national guidelines at the time, patients did not proceed to further therapy.

### Serum sampling and analysis of 25(OH)D

Serum samples were collected at baseline (*n* = 84), immediately following completion of the neoadjuvant therapy (post-Rx; *n* = 63), and at the time of evaluation (*n* = 60; Fig. 1). Analysis of vitamin D metabolites was undertaken at Haukeland University Hospital (Bergen, Norway) in January 2012 (i.e., after 19–75 months of sample storage at −80°C); serum and plasma 25(OH)D concentrations have shown to be largely unaffected by long-term storage [20, 21]. The assay was based on liquid chromatography-mass spectrometry (Agilent Technology, Santa Clara, CA) implementing modifications of the originally described method [22]. The mean recovery of 25(OH)D was 77.2% [standard deviation (SD) 3.9%] with intra-assay variation of 4.9% and detection limit at < 4 nmol/L. Ergocalciferol (vitamin D_2_) can be prescribed as general vitamin D supplementation, and nine patients presented measurable values of its hydroxylated metabolite 25(OH)D_2_, which is considered equipotent with 25(OH)D_3_. Hence, for each of the study patients, the sum of serum levels of 25(OH)D_3_ and 25(OH)D_2_, for practical reasons designated 25(OH)D, was used for analysis.

### Seasonal categorization

Patients’ places of residence were in southern Norway at latitude 58–62°N with a significant variation of solar ultraviolet radiation from one to the next of four seasons [7]. Cases were therefore categorized in four season groups: winter (1 December through 28/29 February), spring (1 March through 31 May), summer (1 June through 31 August), and fall (1 September through 30 November). The patients represented a standard Scandinavian population, the majority of which was Caucasian in the actual time period, but no registration of ethnicity or recording of vitamin D supplementation was undertaken within the study.

### Tumor volumetry

For each patient, on the diagnostic magnetic resonance imaging, the tumor boundary was manually contoured by the study radiologist, and the tumor volume was calculated as previously described [23, 24].

### Treatment outcome measures

The resected tumor specimens were prepared in accordance with the requirements of a validated protocol [25] and histologically evaluated for treatment response according to standard staging (ypTN). Regarding PFS and OS, data were censored on 8 August 2013, at which time 68% of patients had follow-up time of 5 years or longer.

### Statistical analyses

All 25(OH)D measures had normal distribution and seasonal variation was tested with general linear models. The possible influence of factors other than season on the observed change in 25(OH)D measure during the neoadjuvant period for an individual patient was examined by a mixed-effect model with periodic cubic splines according to the visually explored development of 25(OH)D over time, with random intercepts for patients and fixed effects for treatment period. Demographic and clinical characteristics were expressed as median and range, mean ± SD, or percentages in descriptive analyses and as mean ± standard error (SE) in estimate analyses. Baseline tumor and lymph node stages and stages of histologic treatment response in the surgical specimens were dichotomized according to prognostic value. Continuous data with skewed distribution were log-transformed. PFS was calculated from the time of study enrollment to the day of recurrent disease (diagnosis of local recurrence or distant metastasis), death of any cause, or end of follow-up (a maximum of 5 years after the date of surgery or at final censoring), whichever occurred first. OS was measured from the date of enrollment to death of any cause or final censoring. Univariable association analyses were described by the results of independent-samples *t*-test, one-way analysis of variance, and Pearson correlation test, as appropriate, and adjusted for season by linear and logistic regression models. Associations between 25(OH)D levels and PFS or OS were analyzed with Cox proportional hazards models, and results were expressed as hazard ratio (HR) with 95% confidence interval (CI). Age, sex, body mass index (BMI), and the season of serum sampling, which might influence the 25(OH)D level or disease outcome, were adjusted for in the multivariable models. Winter/spring and summer/fall were used as collapsed categories in order to avoid violation of the models by small sample sizes. All statistical analyses were two-sided, and *p*-values less than 0.050 were generally used. Following Bonferroni correction for multiple testing of 25(OH)D and circulating markers possibly related to tumor immune responses (12 analyses), the *p*-value for significance was set to 0.0042 (0.050 divided by 12). The mixed effect-model was performed in R using the lme4 and pbs packages [26]. All other analyses were carried out using STATA version 15 (StataCorp LCC, College Station, TX).

## Results

### Seasonal variation in serum 25(OH)D level

**Fig. 2:**
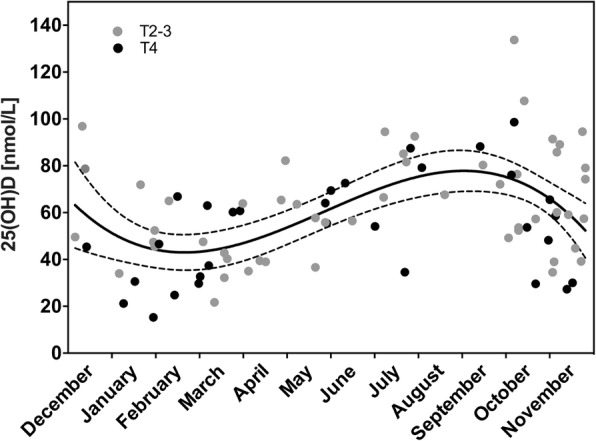
Baseline serum level of 25-hydroxyvitamin D [25(OH)D], the time of the year of study enrollment, and tumor (T) stage for each patient. The fit standard curve (solid line) with 95% confidence bands (dashed lines) for the entire study population is shown

**Fig. 3:**
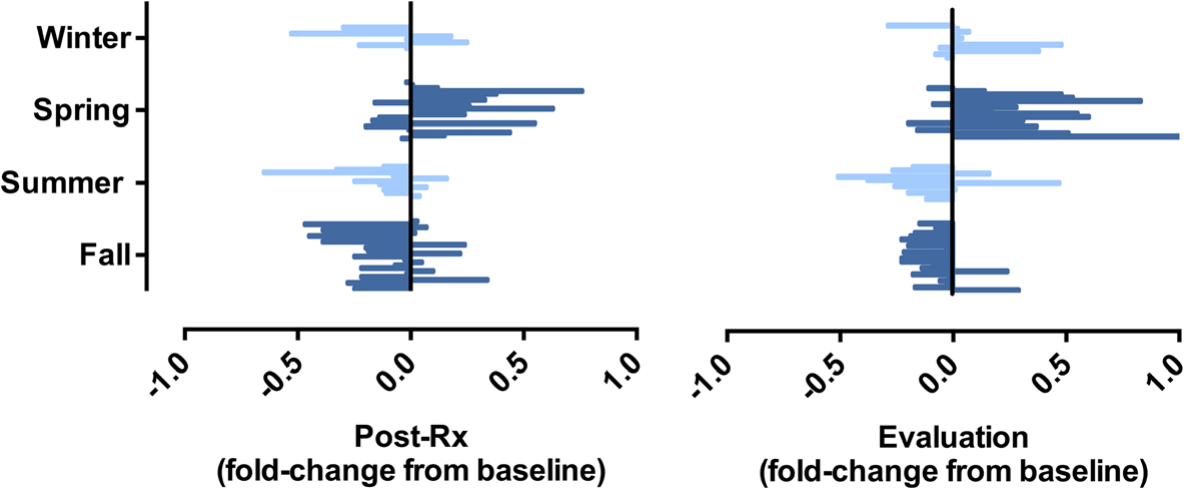
The relative change of serum 25-hydroxyvitamin D level over the neoadjuvant course for individual study subjects. Each patient is represented by a line and assigned a group according to the season of diagnosis. *Abbreviation: Post-Rx* completion of neoadjuvant therapy

Figure 2 illustrates baseline serum 25(OH)D in the individual patients at the given time of diagnosis. Levels (mean of 59.1 ± 22.7 nmol/L within the range of 15.3–133.7 nmol/L for the entire population; *n* = 84) were significantly different among the four season categories (*p* = 0.0017); mean was 49.5 ± 5.3 nmol/L in winter (*n* = 16), 48.7 ± 4.5 nmol/L in spring (*n* = 22), 71.2 ± 5.6 nmol/L in summer (*n* = 14), and 65.8 ± 3.7 nmol/L in fall (*n* = 32). Figure 3 depicts the relative change over the neoadjuvant course for individuals who had baseline and either post-Rx or evaluation measures, as categorized by season of diagnosis. For the actual cases (*n* = 63 and *n* = 60, respectively), median time from baseline to post-Rx serum sampling was 68 (range, 62–90) days and 98 (range, 89–126) days to evaluation sampling. Again, significant seasonal differences were observed within both datasets (*p* = 0.00072 and *p* = 0.00013, respectively). Specifically, 25(OH)D fold-change (mean ± SE) from baseline to post-Rx or to evaluation was −0.11 ± 0.09 and 0.05 ± 0.06 in the winter category, 0.17 ± 0.06 and 0.34 ± 0.09 in the spring category, −0.14 ± 0.07 and −0.12 ± 0.08 in the summer category, and −0.11 ± 0.04 and −0.10 ± 0.03 in the fall category. Hence, a majority of patients diagnosed in winter and spring experienced elevation in serum 25(OH)D over the neoadjuvant period; correspondingly, a decline occurred in most patients diagnosed in summer and fall. After season adjustment for each case by the linear-mixed model, however, only minor alterations in serum 25(OH)D were observed from baseline to post-Rx (mean increase of 0.58 units for the entire cohort; *p* = 0.24) and evaluation (mean decrease of 2.1 units for the entire cohort; *p* = 0.75), indicating that the neoadjuvant treatment as such may not have affected circulating 25(OH)D. The baseline 25(OH)D measure for each patient was therefore used in all of the subsequent analyses.

### Serum 25(OH)D level and disease presentation

As shown in Table 1, 25(OH)D was not correlated with age, sex, or BMI. Low 25(OH)D was significantly correlated with reduced performance status (ECOG 1; *p* = 0.031) and anemia (*p* = 0.004). Interestingly, the mean 25(OH)D measures in patients with these unfavorable clinical features were just below the lower reference interval limit for maintenance of a normal bone metabolism (50 nmol/L) [12]. Moreover, the patient group with T4 disease had significantly lower 25(OH)D (52.5 ± 3.9 nmol/L) than T2–3 cases (63.0 ± 3.1 nmol/L; *p* = 0.040) and similarly, there was an inverse correlation between 25(OH)D value and tumor volume (*r* = −0.267; *p* = 0.025). All of these associations were maintained after season adjustment, with ECOG status as the statistically strongest factor (*p* = 0.015). Furthermore, a separate analysis showed no association between season alone and any of these features (not shown). Ten patients with T4 disease (32.3%) had particularly low 25(OH)D (below 35 nmol/L; Fig. 2). As listed in Table 2, no correlations were seen between serum 25(OH)D and circulating levels of calcium, albumin (the main calcium-binding protein), or carcinoembryonic antigen (a CRC tumor marker). Analyses indicated inverse correlations between the 25(OH)D and common markers of systemic inflammation (C-reactive protein, erythrocyte sedimentation rate, and neutrophil-to-lymphocyte ratio), but statistical significance did not persist after correction for multiple comparisons.

**Table 1:** Serum content of vitamin D and correlations with patient and disease factors

		*n* (%)	25(OH)D (mean ± SE), nmol/L	*r*	*p* ^a^	*p* ^b^
Median age (range), years	58.5 (30–73)	84 (100)		0.079	0.48	0.45
Sex	Female	34 (40.5)	60.2 ± 4.2			
	Male	50 (59.5)	58.4 ± 3.1		0.72	0.76
BMI (mean ± SD), kg/m^2^	24.1 ± 3.5	83 (98.8)		−0.046	0.68	0.60
ECOG	0	67 (79.8)	61.8 ± 2.7			
	1	17 (20.2)	48.5 ± 5.6		0.031	0.015
Hemoglobin	Normal	63 (75.0)	63.2 ± 2.7			
	Anemic^c^	21 (25.0)	47.0 ± 5.1		0.004	0.029
Median tumor volume (range), cm^3^	16.7 (1.0–293)^d^	71 (84.5)		−0.267	0.025	0.024
T stage	2–3	53 (63.1)	63.0 ± 3.1			
	4	31 (36.9)	52.5 ± 3.9		0.040	0.028
N stage	0	11 (13.1)	62.1 ± 7.7			
	1–2	73 (86.9)	58.7 ± 2.6		0.65	0.95
ypT stage^e^	0–3	66 (78.5)	62.4 ± 2.7			
	4	17 (20.2)	48.1 ± 5.6		0.019	0.018
ypN stage^e^	0	56 (66.7)	61.2 ± 3.3			
	1–2	27 (32.1)	55.9 ± 3.5		0.33	0.21

Abbreviations: *25(OH)D* 25-hydroxyvitamin D, *BMI* body mass index, *ECOG* Eastern Cooperative Oncology Group performance status, *SD* standard deviation, *SE* standard error, *TN* tumor-node, *yp* histologic response to neoadjuvant therapy

^a^Described by Pearson correlation test or independent sample *t*-test

^b^Estimated by linear and logistic regression models, with winter/spring and summer/fall as collapsed categories

^c^Anemia was defined as hemoglobin level below the lower reference interval limit used in Norway (< 11.7 g/dL for women and < 13.4 g/dL for men)

^d^The data were log-transformed before analysis

^e^One patient died unexpectedly before pelvic surgery; as a consequence, histologic tumor response data was missing, and the single case was omitted from these analyses

**Table 2:** Serum content of vitamin D and correlations with other circulating factors^a^

	*n* (%)	*r*	*p* ^b^
CEA	84 (100)	0.071	0.52
Calcium	82 (97.6)	0.202	0.069
Albumin	84 (100)	0.176	0.11
CRP	70 (83.3)	−0.285	0.017^c^
ESR	75 (89.3)	−0.315	0.006^c^
Leukocytes	84 (100)	−0.163	0.14
Neutrophils	84 (100)	−0.207	0.059
Lymphocytes	82 (97.6)	0.109	0.33
NLR	82 (97.6)	−0.237	0.032^c^
Monocytes	81 (96.4)	0.025	0.83
Thrombocytes	84 (100)	−0.043	0.70
LDH	84 (100)	0.150	0.17
Creatinine	84 (100)	0.180	0.10

Abbreviations: *CEA* carcinoembryonic antigen, *CRP* C-reactive protein, *ESR* erythrocyte sedimentation rate, *LDH* lactate dehydrogenase, *NLR* neutrophil-to-lymphocyte ratio

^a^All measures of circulating factors but 25-hydroxyvitamin D were log-transformed before analysis

^b^Estimated by Pearson correlation test

^c^Significant *p*-value did not persist after correction for multiple comparisons of 12 datasets on circulating markers possibly related to tumor immune responses, which excluded that on CEA

### Serum 25(OH)D level and treatment effects

As further shown in Table 1, patients who obtained poor tumor response to the neoadjuvant treatment (ypT4) had lower baseline 25(OH)D (48.1 ± 5.6 nmol/L) than ypT0–3 cases (62.4 ± 2.7 nmol/L; *p* = 0.019, maintained after season adjustment). In practice, only T4 cases may obtain a ypT4 outcome. Hence, the data indicated that the neoadjuvant treatment in patients with T4 disease and 25(OH)D measures below the defined sufficiency for normal bone metabolism [12] resulted in ypT4 (i.e.*,* lack of response), in contrast to T4 patients achieving ypT0–3 (i.e.*,* response) who presented 25(OH)D identical to T2–3 cases. In other words, T4 patients (*n* = 30, as one patient unexpectedly died before pelvic surgery) apparently consisted of two groups of subjects in regard to circulating 25(OH)D—one (*n* = 13) that was biologically similar to T2–3 cases and the other (*n* = 17) with therapy-resistant disease. In contrast to ypT stage, no correlation was found between 25(OH)D and residual lymph nodes in the surgical specimen.

As shown in Table 3, when censored, 31 individuals (36.9% of the study population) had experienced a PFS event. Median time to an event was 13.3 (range, 1.2–40.1) months. Four patients had local recurrence as the first event and 27 patients had distant organ disease. Furthermore, 15 deaths (17.9% of the population) were recorded, with median time to death of 32.8 (range, 1.2–68.3) months and median follow-up time for participants still alive of 74.5 (range, 41.2–94.0) months. Motivated by the findings pertaining to serum 25(OH)D and T and ypT stages, subjects with T2–3 and T4 disease were separately stratified according to baseline 25(OH)D above or below 50 nmol/L for survival analyses. By this, no significant association was found between 25(OH)D and either PFS or OS in T2–3 cases; however, a higher PFS percentage was observed for the vitamin D-high cases. For patients with T4 disease and sufficient 25(OH)D, the percentage of recorded PFS events (six of 17 cases; 35.3%) was equal to that of the corresponding T2–3 group (12 of 35 cases; 34.3%). In contrast, T4 patient with insufficient 25(OH)D had almost twice as many PFS events (nine of 14 cases; 64.3%), which translated into a heightened risk compared to T4 patients with sufficient vitamin D status (HR 3.09, 95% CI 1.01–9.45; *p* = 0.048 when adjusted for age, sex, BMI, and season). Similarly, the vitamin D-low cases in the T4 group had considerably enhanced risk of death (HR 17.6, 95% CI 1.99–155; *p* = 0.010 in the adjusted model).

**Table 3:** Serum content of vitamin D and correlations with survival endpoints

	T2–3 cases			T4 cases		
25(OH)D, nmol/L	< 50	≥50	*p* ^a^	< 50	≥50	*p* ^a^
PFS						
No. at risk	18	35		14	17	
No. of events	4	12		9	6	
Univariable HR (95% CI)	0.626	Referent	0.42	2.53	Referent	0.080
	(0.202–1.94)			(0.895–7.12)		
Multivariable HR (95% CI)^b^	0.720	Referent	0.59	3.09	Referent	0.048
	(0.220–2.38)			(1.01–9.45)		
OS						
No. at risk	18	35		14	17	
No. of events	2	5		7	1	
Univariable HR (95% CI)	0.714	Referent	0.69	10.8	Referent	0.026
	(0.180–3.69)			(1.33–88.3)		
Multivariable HR (95% CI)^b^	0.783	Referent	0.79	17.6	Referent	0.010
	(0.130–4.84)			(1.99–155)		

Abbreviations: *25(OH)D* 25-hydroxyvitamin D, *CI* confidence interval, *HR* hazard ratio, *OS* overall survival, *PFS* progression-free survival, *T* tumor stage

^a^Estimated by Cox proportional hazards models

^b^Adjusted for age, sex, body mass index, and season (winter/spring and summer/fall as collapsed categories)

## Discussion

In LARC patients residing at latitude 58–62°N, circulating 25(OH)D levels reflected the season of diagnosis, displaying a mean group measure 50% higher in summer compared to spring, and changed during the well over 3-month period till treatment evaluation essentially owing to season alone. As group, patients who presented organ-invasive tumor showed a mean serum concentration above 50 nmol/L, but a third of T4 cases had strikingly low 25(OH)D (below 35 nmol/L). Individuals who presented T4 disease that did not respond to the neoadjuvant treatment (ypT4 cases) and systemic manifestations in terms of reduced performance status and anemia, translating into unfavorable survival outcome, had a group mean level just below 50 nmol/L. This was notably different from T4 cases with response (ypT0–3), who presented levels identical to T2–3 cases (mean above 60 nmol/L). Our results indicate that serum content of 25(OH)D below the lower limit of the reference interval (50 nmol/L) impacts unfavorably, or simply reflects unfavorable rectal cancer biology and treatment outcome.

Due to the spectrum and intensity variation of ultraviolet B in solar radiation reaching the Nordic countries, inhabitants present an annual fluctuation of circulating 25(OH)D with roughly 50% higher serum concentrations in late summer and early fall compared to winter and spring [9, 27]. This has also been reported for CRC patients [9, 10] and was found again in the present cohort. Following adjustment for change in season, study patients’ individual 25(OH)D levels over the time from diagnosis to treatment evaluation did not seem to be affected otherwise. The observed variations in circulating 25(OH)D owing to season only are in accordance with a study on Norwegian patients with multiple sclerosis, where the predicted time-adjusted value of serum 25(OH)D for the individual patient was relatively stable [28].

Antitumor effects of the active vitamin D metabolite, calcitriol, or synthetic analogs have been extensively examined in experimental models [29–31] and for CRC, such effects have been shown to implicate cell proliferation and differentiation, apoptosis, and immune modulation [32–34]. Hence, our findings of inverse correlations between the 25(OH)D level and tumor stage and volume may essentially reflect biological effects. However, it is not possible from our data to determine whether the poorer patient performance status and anemia were results of low 25(OH)D level per se, or if these patient features were instead concurrent but mutually independent manifestations of the high-risk disease in this particular subgroup of T4 cases. The absence of a clear correlation between circulating 25(OH)D and systemic inflammation markers may serve as an argument against the former. In this regard, interventional studies may provide causal evidence.

Our findings that patients with T2–3 or T4 disease who obtained the intended treatment surrogate responses had 25(OH)D levels well above the lower limit of the reference interval, suggest that the 25(OH)D concentration must be of a certain magnitude to possibly act synergistically with cytotoxic agents. Preclinical studies have demonstrated that vitamin D analogs enhance the effect of chemotherapy and radiation in breast and non-small cell lung carcinomas through mechanistic interactions [35–37]. In a retrospective review of high-risk breast cancer patients receiving neoadjuvant chemotherapy, subjects with vitamin D supplementation had significantly longer disease-free survival than those without [38]. The recently reported prospective SUNSHINE study in patients with metastatic CRC on first-line systemic therapy revealed that those randomized to high-dose vitamin D supplementation experienced longer PFS than the control group patients given a standard vitamin D dose [39]. Finally, observational studies and meta-analyses have shown that high circulating vitamin D concentrations may lower CRC risk [40, 41] and improve prognosis [4, 42–46]. Within this frame of reference, the most noteworthy observation in our study is that a considerable number of patients with organ-invasive disease within the pelvic cavity, which is regarded as the ‘ugly’ rectal cancer entity [17], obtained tumor down-staging and surprisingly good clinical outcome—when presenting with circulating 25(OH)D levels like individuals with T2–3 disease. Regarding the lack of correlation between 25(OH)D and residual lymph nodes in the surgical specimen, it might be that a favorable stromal reprogramming of the tumor microenvironment by vitamin D, as has been demonstrated in experimental pancreatic cancer models [47], does not occur in lymphatic tissues.

There are evident limitations of this post hoc study. First, the cohort is small and results must be interpreted cautiously. On the other hand, more than a third of patients had T4 disease, which firstly is uncommon in reported LARC studies and secondly represented a balanced vitamin D-low versus vitamin D-high population. Next, the analyses reported here had not been planned at the time of trial conduct. Consequently, the observed associations between circulating 25(OH)D and disease presentation and outcome are likely confounded by diet and lifestyle factors, which were not recorded. For instance, occasional sun exposure due to traveling to sunny destinations in darker months is common among citizens of Norway, possibly resulting in less seasonal variation than could be expected for persons living at the actual latitudes. Nevertheless, adjustment for season confounding of the repeat serum 25(OH)D measures over the neoadjuvant period revealed that each individual patient’s level was remarkably stable. Finally, the study did not have a separate validation cohort, which might be difficult to identify because of the geographic relevance.

## Conclusions

The data in this report indicate that curative-intent neoadjuvant therapy in LARC is less likely to succeed if circulating 25(OH)D is below 50 nmol/L, independent of season, in cases with organ-invasive disease at presentation. Hence, a prospective study with vitamin D supplementation during neoadjuvant therapy may provide evidence for causal effects, if existing.

## Abbreviations

25(OH)D_3_: 25-Hydroxyvitamin D_3_
BMI: Body Mass Index
CI: Confidence Interval
CRC: Colorectal Cancer
ECOG: Eastern Cooperative Oncology Group
HR: Hazard Ratio
LARC: Locally Advanced Rectal Cancer
OS: Overall Survival
PFS: Progression-Free Survival
Post-Rx: Completion of Neoadjuvant Therapy
SD: Standard Deviation
SE: Standard Error
TN: Tumor-Node
yp: Histologic Response to Neoadjuvant Therapy
